# Intraoperative fentanyl in endoscopic procedures and their impact on PACU time and costs

**DOI:** 10.1186/s13741-025-00515-x

**Published:** 2025-03-20

**Authors:** Miho Akabane, Kelsey Kukuruza, Timothy Angelotti, Subhas Banerjee, Kazuo Ando

**Affiliations:** 1 Stanford University School of Medicine, Stanford, CA USA; 2 Department of Anesthesiology, Perioperative and Pain Medicine, Stanford University School of Medicine, Stanford, CA 94305 USA

**Keywords:** Postanesthesia care unit (PACU), Endoscopy, Fentanyl, Pain, Anesthesiology

## Abstract

**Background:**

Extended stays in the postanesthesia care unit (PACU) pose challenges in high-volume endoscopies. This study investigates the impact of intraoperative fentanyl use on PACU duration, postoperative pain, and financial implications in outpatient endoscopy.

**Method:**

A retrospective analysis of upper/lower endoscopies at our facility (2020–2022) was conducted, focusing on the relationship between fentanyl use, PACU duration, and pain scales. Financial impacts were also assessed.

**Results:**

Among 11,488 patients, 5787 (50.4%) received intraoperative fentanyl, and 5225 (45.5%) had a long stay at PACU (> 50 min). A larger proportion of patients in the long-stay group (> 50 min) received fentanyl (56.3% vs. 45.4%, *P* < 0.01), and they reported higher Numeric Rating Scale (NRS) pain scores (> 5 in 3.6% vs. 1.2%, *P* < 0.01). The median PACU time was longer for fentanyl recipients (52 vs. 48 min, *P* < 0.01). Multivariable analysis identified fentanyl use, older age, and higher ASA scores (≥ 3) as significant factors for prolonged PACU durations. Fentanyl did not significantly reduce postoperative pain (scores > 5: 2.8% for fentanyl users vs. 2.2% for nonusers). Furthermore, most patients reported no pain post-surgery (93.0% for fentanyl users vs. 95.2% for nonusers). Fentanyl recipients did not have shorter PACU stays within any pain scale category. Financial simulations suggest that fentanyl-free anesthesia management could notably decrease the financial burden within endoscopy operations. Specifically, our institution could have realized an annual saving of at least US $100,308.

**Conclusion:**

Intraoperative fentanyl increases PACU duration by approximately 4 min per patient in endoscopies, without markedly improving pain management. Avoiding fentanyl could lead to significant time and cost savings.

## Key points

Fentanyl is frequently used in anesthesia for endoscopic procedures; however, the impact of fentanyl on postanesthesia care unit (PACU) stays has not been thoroughly investigated. Our findings reveal that the use of fentanyl prolongs PACU stays by 4 min without improving postoperative pain scores. While 5 min may seem short, when considering the high volume of procedures, the use of fentanyl significantly influences hospital management.

## Introduction

Effective patient flow is fundamental to the smooth functioning of hospitals. It hinges on factors such as patient admission and discharge rates, efficient patient transportation, and the complexity of the services provided (Dexter and Tinker 1995; Sinclair 2011). When the post-anesthesia care unit (PACU) is full, it leads to a queue of patients who are waiting to be transferred out of the operating/procedure room after their surgery, which strains hospital resources (Lalani et al. 2013). Lack of a free PACU bed results in delayed room turnover and delays in subsequent surgery cases. Particularly, for rapid endoscopic procedures such as upper and lower endoscopies, which are performed at a high volume and have a high turnover rate, an extended PACU stay after procedures becomes a significant issue in hospital management. One of the potential contributors to this extended stay is delayed emergence from anesthesia leading to an extended anesthetic recovery time (Beaussier et al. 2002).

The practice of anesthesiology has been significantly impacted by the adoption of synthetic opioid for pain control (Brownstein 1993). Traditionally, there has been an understanding among anesthesiologists about the necessity of opioid for intraoperative anesthesia. Yet, opioid come with a range of side effects such as respiratory depression, dizziness, nausea, pain centralization, immunosuppression, postoperative hyperalgesia, and constipation (Le Merrer et al. 2009; Angst et al. 2003; Galvagno et al. 2017). These can potentially extend a patient’s hospital stay or even necessitate further medical intervention. With growing apprehensions regarding these side effects, there is a rising interest in opioid-sparing anesthetics (Bell et al. 2022). Some studies, including randomized controlled trials, indicate that post-surgery pain levels remain comparable regardless of intraoperative opioid administration (Bell et al. 2022; Soffin et al. 2019; Doleman et al. 2018).

In Endoscopic setting, clinical studies have demonstrated that the combination of propofol and fentanyl provides effective sedation with a lower risk of respiratory events compared to propofol alone. However, impact on postoperative outcome is inconclusive (Shin et al. 2014; Cohen et al. 2007; Santos 2013).

Notably, our study found that over 50% of patients received fentanyl, a finding that warrants further exploration. This high utilization raises questions about the rationale behind its administration. While it may be used for pain management, it is also possible that fentanyl is given to reduce propofol exposure, particularly in sicker patients, or to blunt the physiological responses to noxious stimuli during procedures. Understanding these factors is crucial, especially since the vast majority of simple endoscopic procedures do not typically cause significant post-procedural pain.

We hypothesize that extended PACU durations may have significant safety implications for patients, including increased risk of complications, prolonged recovery times, and potential delays in subsequent procedures. By addressing these concerns, we aim to highlight the importance of optimizing PACU flow and minimizing unnecessary delays in patient recovery. Although various models have been suggested for PACU modelling, none have considered the variable of intraoperative fentanyl use (Schoenmeyr et al. 2009; Bhat 2008; Ruiz-Patiño et al. 2017).

This study aims to investigate the relationship between intraoperative fentanyl use, postoperative pain levels, and the duration of PACU stays at a high-volume single center. Furthermore, we seek to examine the financial implications of administering intraoperative fentanyl.

## Methods

### Study population

This study was conducted using data from a retrospectively collected database maintained at Stanford University Medical Center, CA, USA, for the quality improvement project and approved by the Institutional Review Board (protocol number is 72,022). The requirement for informed consent was waived by the Institutional Review Board as the study involved preexisting data without patient names and medical record numbers. The database contains records from January 1, 2020, to December 31, 2022. To begin, we pulled data on patients who had elective upper or lower endoscopic procedures under the supervision of an anesthesiologist at outpatient clinics, specifically those performed with monitored anesthesia care (MAC). We focused on patients with an American Society of Anesthesiologists (ASA) score ranging from 1 to 3. We excluded data from patients who were hospitalized; underwent emergency/urgent procedures; had intubation with tube/laryngeal mask airway; had complicated procedures such as endoscopic retrograde cholangiopancreatography and esophageal manometry; were given ketamine, alfentanil, and hydromorphone during the operation; were preoperatively given fentanyl; or exhibited apparent pain complaints. Only records that included the duration of the PACU stay were considered for this study.

In our facility, we operate with seven procedural rooms. The staffing models are flexible and vary by day; on some days, we supervise certified registered nurse anesthetists, while on other days, we work alongside residents or operate solo. This adaptability in staffing ensures that we can effectively meet the demands of our patient population.

Patients undergoing endoscopic procedures recover in a dedicated PACU specifically designed for these cases. The PACU is staffed by a dedicated team of registered nurses who are trained to monitor and provide care during the recovery period, ensuring a safe and efficient transition from anesthesia to recovery.

The primary outcome was to assess PACU length of stay (LOS) in patients who did and did not receive intraoperative fentanyl. The secondary outcome was to compare postoperative pain in patients who did and did not receive intraoperative fentanyl.

### Power analysis

An a priori power analysis was conducted using the “pwr” package in R to determine the required sample size. Based on an expected effect size of 0.5, an alpha level of 0.05, and a desired power of 0.80, the analysis indicated that a minimum of 64 participants per group would be required to detect significant differences. This sample size was achieved in our study, as the total population consisted of 11,488 patients, ensuring that our conclusions are reliable.

### Statistical analyses

Statistical analyses were conducted using R 4.3.1 (https://cran.r-project.org/). Patient demographics were documented, reporting the frequencies of various characteristics as percentages, alongside median values and interquartile range (IQR). Differences between categorical values were estimated using the chi-square test. Differences between continuous values were assessed with the Mann–Whitney U- or Kruskal–Wallis tests as appropriate. For this study, we defined a long PACU stay as anything over 50 min, based on the median value from our data. We used the optimal matching algorithm to match patients who received intraoperative fentanyl with those who did not. The optimal matching algorithm seeks to minimize the total distance across all matched pairs, ensuring the best possible matches based on the propensity scores. Balance between groups before and after matching was assessed using standardized mean differences (SMDs) and visualized using a Love plot.

The time spent in the PACU was determined by the interval between entering the PACU after surgery and departing from the PACU. In assessing the relationship between PACU stay duration and fentanyl use based on the pain scale, propensity score matching (PSM) was also performed to adjust for potential confounding factors. Upon entering the PACU post-surgery, patients were evaluated on their pain using the Numeric Rating Scale (NRS) on a scale of 0 to 10, with 0 indicating no pain and 10 representing extreme pain (Hartrick et al. 2003). In all the analyses, statistical significance was established below a*p*-value of 0.05.

## Results

### Study population

During the 2020–2022 period, a cohort of 11,488 patients was identified. Out of these, 5787 (50.4%) received intraoperative fentanyl (the median dosage of fentanyl administered during the procedures was 100 μg), and 5701 (49.6%) did not receive any intraoperative fentanyl. The differences in characteristic between patients with longer PACU stay and those with shorter stays were investigated (Table 1). Of the entire cohort, 5225 patients had a long stay at PACU. The group with longer stays had a significantly higher median age (63 vs. 59 years, *P* < 0.01), and a larger percentage had higher ASA scores (*P* < 0.01). There was a significantly higher number of patients who received intraoperative fentanyl in the group with longer stays (56.3 vs. 45.4%, *P* < 0.01). Furthermore, the NRS was significantly higher in the long-stay group, with scores above 5 being reported by 3.6% of these patients compared to just 1.2% in the short-stay group (*P* < 0.01). However, the median operative time was consistent across both groups, standing at 20 min.

**Table 1:** The characteristics difference according to PACU stay duration

	**PACU stay**		
Median (% or IQR)	Long stay (*N*=5,225)	Short stay (*N*=6,263)	*P*
Age	63 [51–72]	59 [48–69]	<0.01
ASA score			
1	92 (1.8)	195 (3.1)	<0.01
2	2,123 (40.6)	3,298 (52.7)	
3	3,010 (57.6)	2,770 (44.2)	
BMI, kg/m^2^	26.1 [22.6–31.0]	26.3 [22.8–30.9]	0.28
Operative time, minutes	20 [12–31]	20 [12–31]	<0.01
Intraoperative fentanyl use, Yes	2,944 (56.3)	2,843 (45.4)	<0.01
Fentanyl dose, micrograms	100 [50.0–100]	100 [50.0–100]	0.78
Intraoperative NSAID use, Yes	9 (0.2)	22 (0.4)	0.10
Pre/intraoperative acetaminophen use, Yes	8 (0.2)	5 (0.1)	0.38
Pain scale (NRS)			
0–5	3,518 (96.4)	3,413 (98.8)	<0.01
6–10	133 (3.6)	43 (1.2)	

Continuous variables: median [IQR]; Categorical variable: number (%)

For Pain scale, we have missing record 1,574 for long stay and 2,807 for short stay

*Abbreviations.* *ASA* American Society of Anesthesiologists, *BMI* Body mass index, *IQR* Interquartile range, *NRS* Numeric Rating Scale, *NSAID* Nonsteroidal anti-inflammatory drug, *PACU* Post-anesthesia care unit

### Relationship between intraoperative fentanyl use and duration of PACU stay

Among the entire cohort, patients who received intraoperative fentanyl had a noticeably longer median PACU stay (52 vs. 48 min, *P* < 0.01). Both univariate and multivariable regression analyses were conducted to identify factors influencing prolonged PACU stays. As Table 2 highlights, the univariate analysis indicated that intraoperative fentanyl use was significantly associated with longer PACU stays, with a coefficient of 4.27 (95% confidence interval [CI]: 2.77–5.77, *P* < 0.01). This association remained significant in the multivariable analysis, where intraoperative fentanyl use had a coefficient of 3.77 (95% *CI*: 2.19–5.35, *P* < 0.01). Additionally, older age and a higher ASA score (≥ 3) were also found to be associated with extended PACU duration in both univariate and multivariable analyses.

**Table 2:** Multivariable regression analysis of prognostic factors for prolonged PACU stays

	**Coefficients [95% *CI*]**	**Std. error**	***t*-value**	***P***
Age	0.06 [0.00–0.11]	0.03	2.09	0.04
ASA score (ref.: 1)				
2	2.32 [− 3.17–7.80]	2.80	0.83	0.41
3	6.09 [0.53–11.6]	2.84	2.15	0.03
BMI	− 0.07 [− 0.18–0.05]	0.06	− 1.13	0.26
Intraoperative NSAID use	14.4 [− 1.97–30.7]	8.33	1.72	0.08
Intraoperative acetaminophen use	10.4 [− 11.2–32.0]	11.0	0.94	0.35
Intraoperative fentanyl use	3.77 [2.19–5.35]	0.80	4.69	< 0.01

*Abbreviations*: *BMI* Body mass index, *CI* Confidence interval, *NSAID* Nonsteroidal anti-inflammatory drug, *PACU* Postanesthesia care unit, *ref*. reference, *Std. Error* standard error

### Differences in pain scale between groups based on intraoperative fentanyl use

To determine if intraoperative fentanyl use had an impact on postoperative pain levels, the characteristics were compared between groups that received fentanyl during surgery and those that did not (Table 3). There appeared to be a trend where intraoperative fentanyl was more frequently given to patients with higher ASA scores. Importantly, the group that received intraoperative fentanyl did not exhibit a significant reduction in postoperative pain scale (scores above 5: 2.8% for fentanyl-treated vs. 2.2% for non-fentanyl-treated). Furthermore, the vast majority of patients, irrespective of fentanyl administration, reported no pain at all post-surgery (93.0% for fentanyl-treated vs. 95.2% for non-fentanyl-treated). Even after adjusting for potential confounding factors using PSM, the pain scale for fentanyl-treated group was not lower than that for non-fentanyl-treated group (scores above 5: 2.3% for fentanyl-treated vs. 2.2% for nonusers) (Table 3).

**Table 3:** The pre-/post-PSM characteristics difference according to opioid usage

	**Opioid usage**		
Median (% or IQR)	**No** (*N* = 3363)	Yes (*N* = 3744)	*P*
**Pre-PSM**			
Age	62 [50–71]	61 [50–70]	< 0.01
BMI, kg/m^2^	26.2 [22.6–30.9]	26.3 [22.6–31.0]	0.82
ASA score			
1	69 (2.1)	90 (2.4)	< 0.01
2	1642 (48.8)	1587 (42.4)	
3	1652 (49.1)	2067 (55.2)	
PACU stay duration, minutes	48 [37–61]	52 [42–65]	< 0.01
Pain scale (NRS)			
0	3201 (95.2)	3483 (93.0)	< 0.01
1–5	89 (2.6)	158 (4.2)	
6–10	73 (2.2)	103 (2.8)	
Median (% or IQR)	No (***N*** = 2749)	Yes (***N*** = 2749)	*P*
**Post-PSM**			
Age	61 [50–71]	62 [50–71]	0.96
BMI, kg/m^2^	26.2 [22.6–30.9]	26.1 [22.6–31.0]	0.83
ASA score			
1	56 (2.0)	61 (2.2)	0.47
2	1352 (49.2)	1308 (47.6)	
3	1341 (48.8)	1380 (50.2)	
PACU stay duration, minutes	48 [37–61]	52 [42–65]	< 0.01
Pain scale (NRS)			
0	2609 (94.9)	2564 (93.3)	< 0.01
1–5	79 (2.9)	122 (4.5)	
6–10	61 (2.2)	62 (2.3)	

Continuous variables: Median [IQR]. Categorical variable: Number (%)

*Abbreviations:* *ASA* American Society of Anesthesiologists, *BMI* Body mass index, *NRS* Numeric Rating Scale, *PACU* postanesthesia care unit

### Relationship between pain score and duration of PACU stay

**Fig. 1:**
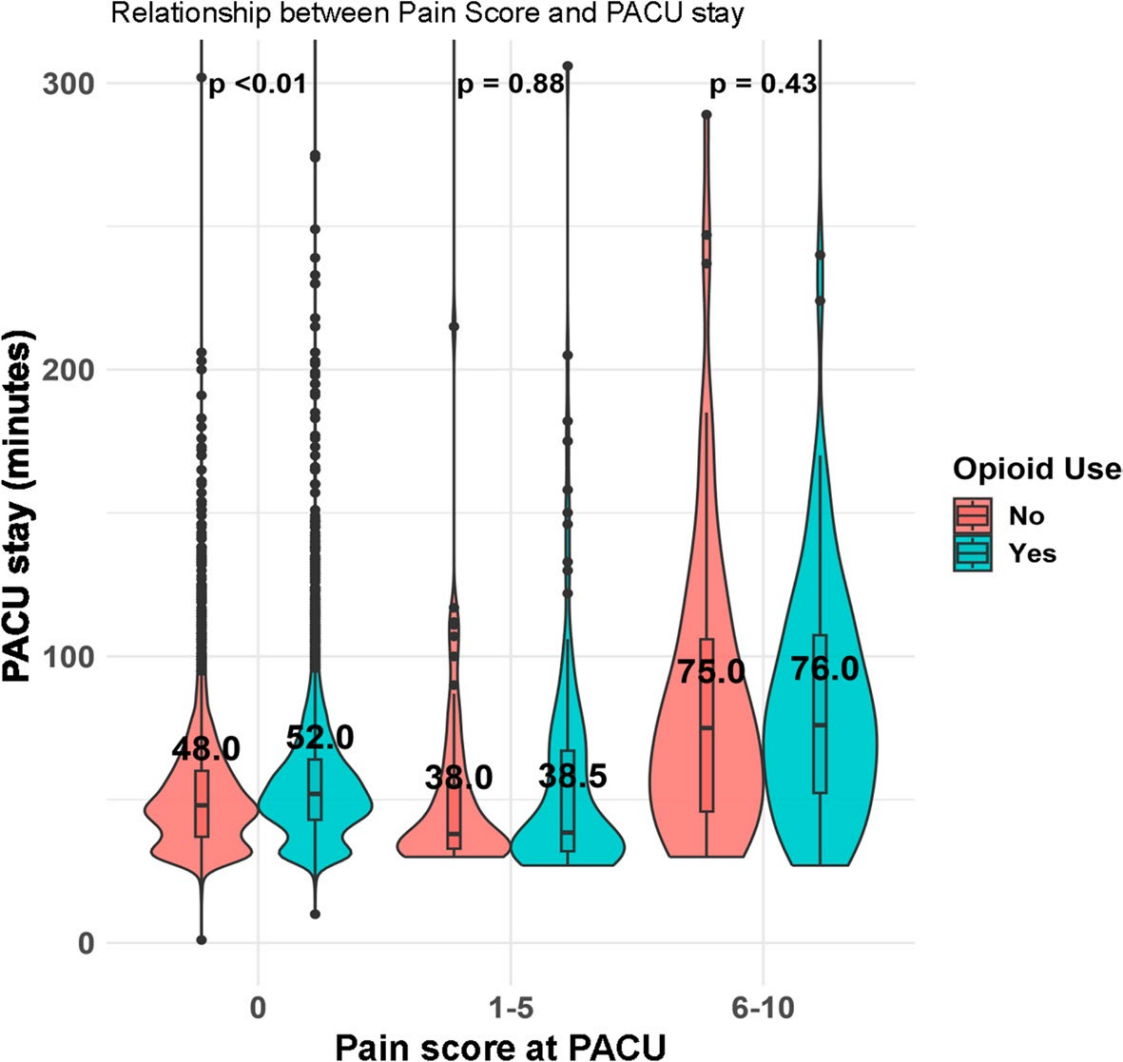
Correlation between postoperative pain score and PACU stay duration

Figure 1 represents the duration of PACU stays corresponding to increasing pain scores. This figure reveals that as pain scales climb above 5, there is a noticeable elongation in the PACU stay duration. Notably, when comparing stays within the same pain scale categories, the group that received intraoperative fentanyl did not exhibit shorter PACU stays. This pattern persists even in categories denoting higher pain scales (pain score = 0: 48 vs. 52 min, *P* < 0.01; 1–5: 38 vs. 38.5 min, *P* = 0.88; 6–10: 75 vs. 76 min, *P* = 0.43).

### Financial implications of intraoperative fentanyl use

According to our analysis after the PSM (Table 3), the use of intraoperative fentanyl leads to an extension of 4 min (52 vs. 48 min, *P* < 0.01) in the PACU. Given prior research estimating the cost of PACU time at roughly US $13 per minute, this extension equates to an added expense of US $52 per case (Raft et al. 2015). Given that our dataset over the 3-year period identifies 5787 cases where intraoperative fentanyl was used, opting to forego this fentanyl in all instances could result in annual savings of approximately US$100,308. In addition to this, the prolonged patient discharge from the PACU may contribute to delays in scheduling endoscopic cases, which, in turn, can have a notable impact on the financial aspects of the operating room (OR). Notably, the cost of OR charges per minutes has been previously estimated at $37 (Childers and Maggard-Gibbons 2018). While we are estimating the costs for the endoscopic suite, it is reasonable to assume that these costs are similar to those of the OR, given the overlap in resource utilization and operational expenses. Improving efficiency within endoscopy suites will contribute to financial benefits.

## Discussion

The present study was conducted to assess the impact of intraoperative fentanyl use on the duration of PACU stays after endoscopic procedures and the broader financial implications for the hospital and the pain outcomes. Our findings indicate that the administration of intraoperative fentanyl acts as a significant independent factor prolonging the PACU stay, extending it by 4 min per case. However, despite the use of fentanyl, there is no notable reduction in the postoperative pain scales. Remarkably, even for those in higher pain scale categories, fentanyl use does not seem to reduce the PACU stay. In fact, the PACU stay durations are almost similar to those who did not receive fentanyl.

The importance of enhancing patient flow during the post-anesthesia phase has long been recognized. In 2005, Dexter and colleagues put forward several strategies to improve patient flow (Raft et al. 2015). The influence of intraoperative opioid use on PACU stay duration remains a topic of debate. This consideration becomes even more crucial for endoscopic procedures, given their sheer volume and the consequent implications for financial management. This analysis was conducted based on a comprehensive database to explore the relationship between intraoperative fentanyl use and PACU time, focusing on endoscopic procedures carried out under MAC anesthesia by anesthesiologists.

The results of our multivariable analysis indicate that intraoperative fentanyl use is a significant risk factor for prolonging the PACU stay, adding an extra 4 min per patient. When considering the financial implications, our institution could have realized an annual saving of at least US $100,308, as per prior studies estimating the cost of PACU time at approximately US $13 per minute (Raft et al. 2015). Also, reducing PACU time by just 4 min per patient can yield an extra 40 min daily, given our facility’s average of 10 cases per room per day. This gain allows for at least one additional procedure per room. If each of the seven endoscopy rooms accommodates one extra procedure, we could perform seven more procedures daily, significantly improving efficiency and revenue.

With our facility billing approximately US $7000 per colonoscopy, this increase could generate an additional US $49,000 per day. When considering these factors collectively, it becomes clear that managing anesthesia without fentanyl has the potential to significantly reduce the financial burden in endoscopy suites.

The real debate lies in the impact of this “4-min” extension. While some may consider it clinically insignificant, the high volume of daily endoscopic procedures means these seemingly minor time savings can accumulate into a substantial duration, leading to significant cost implications.

In a compromised general condition, such as the elderly or those with high ASA scores, typically had extended PACU stays. While it is straightforward to link longer PACU stays with suboptimal pain management, intraoperative fentanyl use did not significantly reduce the PACU stays. Given the pharmacokinetics of fentanyl and the typical duration of the surgical procedures analyzed, fentanyl at a dose of 100 μg has a duration of action of approximately 30–60 min. In our study, the median operative time was 20 min (12–31 min), suggesting that by the time patients reached the PACU, the analgesic effects of fentanyl would have significantly diminished. This observation aligns with the argument that intraoperative fentanyl use does not substantially contribute to postoperative pain relief but rather may extend the PACU stay unnecessarily. Furthermore, an analysis of endoscopic procedure durations revealed no statistically significant differences between the fentanyl-administered group and the non-fentanyl-administered group. This suggests that fentanyl use does not contribute to the amelioration of procedural disruptions, such as patient movement and coughing, during endoscopic procedures. A key observation was that nearly all patients reported a pain scale of 0, regardless of fentanyl administration. This suggests that postoperative pain following endoscopic procedures is generally low and manageable without fentanyl. However, fentanyl is linked to numerous side effects, including respiratory depression and nausea (Fawcett and Jones 2018). These side effects can contribute to longer recovery times, extended PACU stays which elevate healthcare costs (Gan et al. 2014). Given these findings, one might argue that the potential benefits of intraoperative fentanyl are outweighed by their drawbacks. Our data did not reveal any specific patterns for fentanyl use, such as extended operative time, other than higher ASA scores. This may suggest that the decision to administer fentanyl often relies on the individual physician’s judgment. However, due to our mixed model of solo anesthesiologists and resident/CRNA supervision, we are unable to identify patterns to assess variations in practice. It is essential to clarify that advocating for fentanyl-free anesthesia does not mean excluding all pain-relief methods. While our study explored the impact of fentanyl use on PACU duration and hospital finances, it is worth noting the broader societal implications.

The limitations of this study include its retrospective nature and the lack of detailed data on postoperative nausea and vomiting (PONV) and propofol dosage, preventing us from assessing its impact. Another potential confounding factor that could extend PACU stays is delays in retrieving patients, particularly due to our institutional policy requiring an accompanying individual for discharge after anesthesia. Additionally, since this data was collected during the height of the pandemic, visitor restrictions made it challenging to facilitate their discharge home. However, such occurrences are likely consistent, irrespective of fentanyl usage. Given the large sample size of our study, this factor is likely negligible. Understanding and addressing the causes of delayed discharge in PACU may help to improve patient flow and reduce discharge times (Cobbe and Barford-Cubitt 2018). While our study suggests that reducing PACU stay by minimizing intraoperative fentanyl use could have financial benefits, it is important to acknowledge the potential operational challenges associated with this approach. Shorter PACU times have the potential to enhance throughput by enabling the processing of more patients in less time—thereby freeing up resources and reducing wait times for subsequent surgeries—this improvement is contingent upon the alignment of all other system factors, including staffing, bed availability, and OR efficiency. In practice, operational challenges may arise when these components cannot keep pace with the accelerated turnover in the PACU. On the financial side, cost comparisons across different hospitals are challenging due to varying structures. Therefore, our financial estimates should be seen as references rather than definitive figures. The shared resources between the OR and the PACU, such as staff and medications, introduce further complexity in cost calculations (Raft et al. 2015). Nevertheless, our study’s strength lies in its large, relatively homogeneous sample and precise definitions of several key variables. It is crucial to remember that financial costs fluctuate and need regular monitoring.

In conclusion, our findings show that intraoperative fentanyl use extends PACU stays following endoscopic procedures, adding about 4 min per patient. However, fentanyl use does not enhance postoperative pain management. Financially, this practice can result in significant annual time and cost burdens. In the context of endoscopic procedures, avoiding fentanyl might not comprise pain control and could lead to cost savings.

## Data Availability

The data are not publicly available due to patient privacy concerns.
